# Alternative Lengthening of Telomeres in the Budding Yeast *Naumovozyma castellii*

**DOI:** 10.1534/g3.119.400428

**Published:** 2019-08-19

**Authors:** Marita Cohn, Ahu Karademir Andersson, Raquel Quintilla Mateo, Mirja Carlsson Möller

**Affiliations:** Department of Biology, Genetics group, Lund University, 223 62 Lund, Sweden

**Keywords:** ALT, Alternative Lengthening of Telomeres, telomere, genome integrity, telomerase-independent telomere maintenance, yeast, *Naumovozyma castellii*

## Abstract

The enzyme telomerase ensures the integrity of linear chromosomes by maintaining telomere length. As a hallmark of cancer, cell immortalization and unlimited proliferation is gained by reactivation of telomerase. However, a significant fraction of cancer cells instead uses alternative telomere lengthening mechanisms to ensure telomere function, collectively known as Alternative Lengthening of Telomeres (ALT). Although the budding yeast *Naumovozyma castellii* (*Saccharomyces castellii*) has a proficient telomerase activity, we demonstrate here that telomeres in *N. castellii* are efficiently maintained by a novel ALT mechanism after telomerase knockout. Remarkably, telomerase-negative cells proliferate indefinitely without any major growth crisis and display wild-type colony morphology. Moreover, ALT cells maintain linear chromosomes and preserve a wild-type DNA organization at the chromosome termini, including a short stretch of terminal telomeric sequence. Notably, ALT telomeres are elongated by the addition of ∼275 bp repeats containing a short telomeric sequence and the subtelomeric DNA located just internally (TelKO element). Although telomeres may be elongated by several TelKO repeats, no dramatic genome-wide amplification occurs, thus indicating that the repeat addition may be regulated. Intriguingly, a short interstitial telomeric sequence (ITS) functions as the initiation point for the addition of the TelKO element. This implies that *N. castellii* telomeres are structurally predisposed to efficiently switch to the ALT mechanism as a response to telomerase dysfunction.

The ends of linear chromosomes are protected by special DNA-protein complexes, telomeres. Telomeres are essential for both genome integrity and cell proliferation. Every time a cell divides, a piece of DNA is lost due to the “end replication problem’’ (Olovnikov 1973). This progressive attrition of telomeres triggers DNA damage response at chromosome ends, which are not able to distinguish themselves from DNA double-strand breaks. At the crisis point, cellular proliferation will stop and cells will eventually die. Conversely, cancer cells escape from cell cycle arrest and telomere dysfunction triggers genome instability (Kim *et al.* 1994). Hence, telomere-induced cellular senescence could be considered as a “tumor-suppressing mechanism” (Shay and Wright 2011).

Most eukaryotic organisms, including yeasts, counteract this progressive telomere shortening by employing the enzyme telomerase, which is a ribonucleoprotein complex with a specific RNA subunit. Telomerase adds telomeric repeats onto chromosomal ends by using its intrinsic RNA template (Greider 1991). Due to insufficient telomerase activity, human somatic cells undergo a limited number of cell divisions (Hayflick and Moorhead 1961; Harley *et al.* 1990). In contrast, most cancer cells overcome replicative limitations to gain cellular immortality by reactivating telomerase. Even though telomerase is a key component for unlimited proliferation, 10–15% of cancer cells maintain their chromosome ends over many population doublings in the absence of telomerase. These cells use alternative mechanisms rather than re-activating the telomerase. Collectively, the telomerase-independent mechanisms are known as Alternative Lengthening of Telomeres (ALT) (Bryan *et al.* 1997). Although telomerase is an attractive target for anti-cancer therapies, development of an effective treatment will benefit by addressing the ALT pathways (Cesare and Reddel 2010).

Budding yeasts express telomerase and proliferate indefinitely. When telomerase is disabled in *Saccharomyces cerevisiae*, cells divide for a limited number of generations, like human somatic cells. Their telomeres progressively shorten and very short telomeres inhibit cell proliferation (Lundblad and Szostak 1989). Telomerase-negative cells display a decline in growth rate and most cells stop dividing and senesce. A few of them overcome the replicative senescence, improve their growth rate and “survivors” arise (Lundblad and Blackburn 1993). In order to restore telomere lengths they amplify telomeric and subtelomeric sequences via recombination-mediated ALT pathways (Kass-Eisler and Greider 2000; Chen *et al.* 2001).

Recombinational telomere extension has been described also in *Kluyveromyces lactis* (McEachern and Blackburn 1996). It is suggested that telomerase deletion mutants use the “roll and spread” mechanism where a telomeric circle (t-circle) is used as a template to elongate an eroded telomere. This newly elongated telomere is then used as a template to elongate other telomeres through break-induced replication (BIR) (Natarajan and McEachern 2002; Groff-Vindman *et al.* 2005; Topcu *et al.* 2005; McEachern and Haber 2006).

Recombination is actively used but not essential for formation of all types of survivors. A subset of *S. cerevisiae* cells still proliferate in the absence of both telomerase and recombination through formation of palindromes (PAL) (Maringele and Lydall 2004). PAL survivors have been observed when ExoI (exonuclease I) is disabled in *S. cerevisiae* cells lacking both telomerase and recombination. They contain linear but abnormally sized chromosomes without telomeric sequences (Maringele and Lydall 2004). In contrast, when telomeres of *S. cerevisiae* strains were first lengthened, rare survivors exhibiting telomeric DNA amplification appeared in telomerase- and *RAD52*-deficient cells (LeBel *et al.* 2009).

The budding yeast *Naumovozyma castellii* has been used as a model organism in telomere studies owing to its human-like telomere structure and maintenance (Karademir Andersson and Cohn 2017). *N. castellii* belongs to an evolutionary lineage that underwent whole genome duplication followed by a massive gene loss (Cliften *et al.* 2006; Karademir Andersson *et al.* 2017). Its genome is sequenced and has been used extensively in comparative genomics (Karademir Andersson and Cohn 2017). *N. castellii* has ten chromosomes and a genome size of 11.2 Mb (Cliften *et al.* 2006; Gordon *et al.* 2011). Its processive telomerase enzyme is an excellent tool to study molecular mechanisms of telomerase-dependent telomere maintenance (Cohn and Blackburn 1995). The regular octamer repeats, TCTGGGTG, have enabled the determination of the minimal binding sites for the telomere proteins Rap1 and Cdc13 (Cohn *et al.* 1998; Wahlin *et al.* 2003; Rhodin Edsö *et al.* 2008). Recently, we showed that the single-stranded overhang varies in length between 14-200 nt (Fridholm *et al.* 2013).

Here, we report that telomerase-negative *N. castellii* cells avoid replicative senescence, and proliferate without any major growth crisis, by using an ALT mechanism with novel characteristics. The efficient ALT mechanism is observed in both diploid and haploid *tlc1*^-^ mutants, as well as in diploid *est1*Δ mutants. *N. castellii* ALT cells maintain linear chromosomes and lack any gross chromosomal rearrangements. Interestingly, ALT cells maintain a similar chromosomal end structure as the wild-type, but with a shorter telomeric region. Intriguingly, we discovered that ALT telomeres are lengthened by uniform ∼275 bp repeats containing a subtelomeric region and the shorter telomeric region. We propose that telomere lengthening occurs by inter-telomeric copying, starting from a short interstitial telomeric sequence in the subtelomeric element. The actual structure of the *N. castellii* subtelomeric DNA may therefore form the foundation for the efficient activation of this novel type of ALT maintenance mechanism.

## Materials and Methods

### Strains and media

The yeast *Naumovozyma castellii* was previously called *Saccharomyces castellii* or *Naumovia castellii*. The *N. castellii* strains used in this study were NRRL Y-12 630 (type strain), Y235 (*MATa/MATα*, *ura3/ura3)*, YMC121, YMC124, YMC125 and YMC130 (*MATa/MATα*, *ura3/ura3*, *tlc1*::*kanMX3/tlc1*::*kanMX3*); YMC122 and YMC123 (*MATa/MATα*, *ura3/ura3*, *TLC1/TLC1*); YMC300 (*MATa/MATα*, *ura3/ura3*, *EST1/est1Δ*::*URA3*), YMC48 and YMC63 (*MATα*, *hoΔ*::*hphMX4*, *ura3*); YMC481, YMC482 and YMC631 (*MATα*, *hoΔ*::*hphMX4*, *ura3*, *tlc1*::*kanMX3*) (Astromskas and Cohn 2007; Astromskas and Cohn 2009; Karademir Andersson *et al.* 2016). YMC300 (*MATa/MATα*, *ura3/ura3*, *EST1/est1Δ*::*URA3*), YMC303, YMC305 (*ura3/ ura3*, *est1Δ*::*URA3/est1Δ*::*URA3*). *Escherichia coli* strain DH5α was used in the cloning experiment.

All yeast strains were grown in YPD medium containing 1% (w/v) yeast extract, 2% (w/v) peptone and 2% (w/v) glucose. Ura^-^ drop out plates contained 26.7 g/l minimal SD base, 0.77 g/l ura^-^ DO supplement and 2% agar. Geneticin and hygromycin resistant cells were grown in YPD medium containing 75 mg/l geneticin and 200 mg/l hygromycin B (Duchefa), respectively. Yeast cells were grown at 25°.

The *TLC1* gene was disrupted by using the TLC1-kanMX3 construct as previously described (Astromskas and Cohn 2009), with the kanMX3 cassette inserted into the *Hpa*I site of the *N. castellii TLC1* gene. PCR amplification of the plasmid template was performed with primers 5′- ATTTAGTATTTTGTTTTCCCG -3′ and 5′- TGAATGATTTACTTGTCGTCGC -3′ and annealing at 57° for 30 s. To confirm insertion into the correct genomic DNA site of transformants, PCR amplicon size was estimated by using primers annealing 0.5-0.6 kb up- and downstream regions of the *TLC1* gene, respectively: 5′- GTAGTTTCTTCGTCAGCC -′3 and 5′- TTGGCTCCAGAAGTCGC -3′. To further confirm the *tlc1*^-^ genotype, *Pvu*I-treated genomic DNA (gDNA) extracted from spores of five tetrads was analyzed with Southern blot hybridization (previously described in (Astromskas and Cohn 2009)). Using the *TLC1* gene as the hybridization probe, the fragment size difference confirmed the correct insertion of the kanMX3 marker within the *TLC1* gene (Astromskas and Cohn 2009).

The *EST1* gene was deleted in the *N. castellii* strain Y235, leading to strain YMC300. The deletion cassette was constructed by using the plasmid pWJ1042 containing the *K. lactis URA3* gene flanked by 143 nt long direct repeats, allowing the subsequent pop-out of the *URA3* gene (Reid *et al.* 2002; Karademir Andersson *et al.* 2016). The deletion cassette was PCR amplified in two separate fragments with primers: 1) 5′ – TAGTTAAACATATTTACGACACCAACGGTGACCCGATTCTGG- GAATTGGGTACCGGGCCC -3′ and 5′- CTGATATCACCAACGCCC -3′, 2) 5′- GAGCGCTGATTCTCTTTTGG -3′ and 5′- TCAAGATTGATCTAGTTGGATT-ATTGAACATTTTCTGAACAGTGA ATGGCAATTCCCGGGGATC -3′. The 45 nt long up- and downstream regions of the *EST1* gene are underlined and the rest correspond to complementary sequences up- and downstream of the direct repeats in pWJ1042. PCR with Phusion DNA polymerase was performed according to manufacturer’s instruction, with annealing at 50° for 30 s. Correct insertion into transformants was determined by PCR, using primers annealing 0.5 kb up- and downstream of the *EST1* gene, respectively: 5′- ATCTCGGTGCTCAAAGGTGC -3′ and 5′-ACAGCCTTGGCAGATATCGAAG -3′. Verification of knockouts was also performed by Southern blot hybridization of *Bam*HI cleaved genomic DNA, using an oligonucleotide probe specific for the *klURA3* marker gene (5′- CTGATATCACCAACGCCC -3′) which was 5′ end-labeled with [γ^32^P]-ATP by T4 Polynucleotide kinase (NEB). Hybridization conditions as previously described at 40° (Astromskas and Cohn 2009; Karademir Andersson *et al.* 2016).

Both the deletion and the disruption cassettes were transformed using lithium acetate method and yeast cells were spread on appropriate selective plates, as previously described (Astromskas and Cohn 2009; Karademir Andersson *et al.* 2016). Diploid cells were sporulated and tetrads were dissected on YPD plates with the Singer MSM microdissection system according to the manufacturers manual, as previously described (Astromskas and Cohn 2009; Karademir Andersson *et al.* 2016).

### Preparation of Naumovozyma castellii telomerase extract

The *tlc1*^-^ cells (>75 generations) were grown in YPD medium at 25° overnight (ON) and harvested at OD_600_ = 1-1.5. The cells were resuspended in TMG buffer (10 mM Tris-HCl pH 8, 1.2 mM MgCI_2_, 0.1 mM EDTA, 0.1 mM EGTA, 1.5 mM DTT, 40U/ml RiboLock RNasin (Thermo Scientific), 1x Complete Protease inhibitor cocktail (Roche) and disrupted using a bead beater. The protein fraction was ultracentrifuged in a 50.2 Ti rotor (Beckman) at 40 000 rpm, 4° for 90 min. The crude extract was partially purified on a DEAE-agarose column, which was equilibrated with TMG buffer (10% glycerol) and washed with 0.4 M and 0.5 M sodium acetate in TMG buffer (10% glycerol) respectively. The active telomerase enzyme was eluted using 0.6 M sodium acetate in TMG buffer (10% glycerol). Desalting and concentrating was done using Amicon Ultra-15 centrifugal filters (Millipore, 30K MWCO). Aliquots were stored in -80°. All solutions were prepared with Diethylpyrocarbonate treated water to prevent RNase contamination.

### Telomerase primer extension assay

Telomerase activity was assayed in a total volume of 20 μl at 18° for 30 min, with 10 μl of the telomerase extract in a 1x telomerase reaction buffer (0.05 M Tris-HCl pH 8.0, 1mM spermidine, 1mM DTT), 50 μM dTTP, 50 μM dCTP, 200 mM K-Glu, 0.083 μM [α^32^P]-dGTP (PerkinElmer), 0.54 μM dGTP, and a gel purified oligonucleotide, (GGGTGTCT)_2_, with a final concentration 1.65 μM. The primer extension reactions were stopped with 200 μl 10 mM Tris-HCl (pH 7.5) and 21 mM EDTA. An 11-mer telomeric primer, which was labeled with [γ^32^P]-ATP by T4 Polynucleotide kinase, was added into the stop solution as loading control. Extraction was made with an equal volume of phenol/chloroform:isoamyl alcohol (24:1). An equal volume of 5 M ammonium acetate with 0.1 mg/ml glycogen was added before DNA precipitation with 1 ml 95% ethanol in room temperature for 60 min. The samples were washed with 70% ethanol and dried. Pellets were resuspended in 4 μl Xylene-cyanol dye mix, incubated at 90° for 2 min and loaded on a 10% polyacrylamide/7 M Urea sequencing gel. The gel was run at 1800 V for 1h 40 min in 0.6 x TBE buffer (26.7 mM Tris-Borate and 0.6 mM EDTA), dried and analyzed for radioactive signal with a BioRad Molecular Imager FX PhosphorImager.

### Colony streaking assay

After sporulation and microdissection of asci, tetrad spores were germinated and grown into colonies at 25° for 48 hr (passage 1). Colonies were then restreaked onto fresh YPD plates (passage 2). After 48 hr incubation at 25°, single colonies were picked and restreaked again (passage 3). Restreaking was repeated to obtain up to 12-16 subsequent passages. Each passage corresponds to 20-25 generations (Reid *et al.* 2002; Karademir Andersson *et al.* 2016). Several separate streaks were then assembled onto the same plate for the figure preparation.

### Yeast spot test assays

Whole colonies from passage 2-15 (p2-p15) of the diploid *tlc1*^-^ ALT strain, and the isogenic WT strain, were grown overnight in 2 ml YPD, at 25°. Undiluted, and serial 10-fold dilutions of the cultures were spotted onto YPD plates and incubated for 2 days at 25°.

### Liquid growth assays

Colonies from the diploid *tlc1*^-^ strain, passage 3 (p3) and passage 16 (p16), as well as wild-type (WT) strains obtained from the same sporulation tetrad, were inoculated into liquid YPD media and grown overnight at 25°. Next day, cultures were inoculated to a final concentration of 5 × 10^5^ cells/ml. After 24 hr of growth at 25° the cell density was counted in a Bürker chamber. The mean value of ten independent experiments was calculated and plotted together with the standard deviation.

### DNA isolation and terminal restriction fragment assay (TRF assay)

One single colony from each passage was inoculated in 2-10 ml YPD liquid media and grown for ON at 25°, 200 rpm. Cells were mechanically broken using glass beads. DNA extraction was done with phenol/chloroform:isoamyl alcohol and then with ethanol. After RNase A incubation at a final concentration of 75 mg/l, genomic DNA (gDNA) was precipitated with 25 mM NH_4_Ac followed by ethanol. Appropriate amount of gDNA was digested by either *Hind*III or *Dde*I (ThermoScientific). 300 ng of digested gDNA was separated on a 0.8% agarose gel, 0.5 x TBE, and was transferred to a Hybond-XL membrane (Amersham). Hybridization probes were 5′end-labeled by T4 polynucleotide kinase and [γ^32^P]-ATP. The membrane was hybridized with a telomeric probe, (TGTCTGGG)_2_, at 40° for ON. The membrane was washed for 5 min and then washed at 45° for 15 min, twice in 100 mM Na_2_HPO_4_ and 2% SDS. Signals were visualized using Typoon FLA 9500 (GE Healthcare).

### Bal31 assay

1.4 μg gDNA was incubated with 0.3U of Bal31 nuclease (NEB) for increasing time at 30°. In each time point, 1/10 volume of the reaction was taken out and Bal31 was heat-inactivated at 65° for 10 min in the presence of 33 mM EGTA. DNA was phenol-chloroform-extracted, ethanol-precipitated, and treated by either *Hind*III or *Dde*I. Digested DNA was separated on a 0.8% agarose gel (140 ng DNA per well) and subjected to Southern blot analysis as described above. As a control, linear plasmid DNA was incubated with Bal31 and digestion was assessed by a decrease in size of the linear plasmid DNA on an agarose gel (data not shown).

### Pulsed field gel electrophoresis (PFGE)

Yeast cells were grown at 25°, ON, one single colony inoculated into 4 ml YPD. Cells from 1-2 ml samples were pelleted, washed twice in 1 ml Tris/EDTA-solution (10 mM Tris-HCl pH 7.5, 50 nM EDTA) and dissolved in 100 μl Tris/EDTA-solution. Cells were placed at 42° for 30 s and mixed with 950 μl Low-melting-point (LMP) agarose (1% w/v in 125 mM EDTA pH 7.5, 42°). The cell suspension was pipetted into plug molds and allowed to solidify at 4°. Agarose plugs were collected into 2 ml microtubes with 1.5 ml 10 mM Tris-HCl pH 7.5, 0.5 M EDTA. Protoplasts were prepared by adding 40 μl 10 mg/ml Zymolyase (20T, US Biological) and incubated at 37° ON. Next day the buffer was removed and chromosomes were prepared by addition of 1 ml 10 mM Tris-HCl pH 7.5, 0.5 M EDTA, 1% N-lauroylsarcosine pH 9.5, 2 mg/ml Proteinase K and incubation ON at 50°. The plugs were washed five times with 2 ml Tris/EDTA-solution for 1 hr at room temperature and finally stored in Tris/EDTA-solution at 4°. The plugs were inserted in the wells of a 1% agarose gel (Bio-Rad Pulse field certified agarose) in 0.5x TBE and fixed with LMP agarose. Chromosome size marker: *S. cerevisiae strain* YNN295 (Pharmacia). Chromosomes were separated by pulsed field gel electrophoresis (PFGE) employing a CHEF Mapper XA (Bio-Rad), 0.5x TBE at 14°, and then stained in 0.5 μg/ml ethidium bromide, 0.5x TBE, for 2-4 h. Separation program: Block 1, pulse-time 240 s for 8 h; block 2, pulse-time 160 s for 10 h, block 3, pulse-time 90 s for 14 h, block 4, pulse-time 60 s for 8 h; angle 60°, 4.5 V/cm.

### Cloning of telomeric fragments

Genomic DNA of passage 11 (∼275 generations) was digested with *Dde*I and then separated on a 0.8% agarose gel. *Dde*I-treated fragments shorter than 350 bp were gel-extracted. Purified fragments were blunt-end treated with T4 DNA polymerase (Fermentas) according to manufacturer’s instruction. These fragments were spin-purified and then ligated into dephosphorylated *Sma*I-treated pUC18. The ligation mixture was transformed into *E. coli* cells and transformants were selected on LB-ampicillin plates containing X-gal and IPTG. Transformants were passaged onto both a master plate and a Hybond N membrane (Amersham). In total, 10 202 colonies were screened for the presence of telomere sequences by colony hybridization with the telomeric probe. A total of 448 colonies giving hybridization signal were subjected to plasmid DNA (pDNA) purification and were further screened by slot blot hybridization, where the clones showing hybridization signal were selected for sequencing using the universal primer M13 (17-mer). Three colonies (ID 382, ID 406, and ID 448) showed a strong hybridization signal, indicating telomere-containing insert.

### Slot blot analysis

Genomic DNA from ALT cells with and without the ALT ladder pattern was spotted on the membrane. Negative control: λ DNA. Positive control: pDNA of the clone ID 406, which contains both the TelKO element and 27 bp long telomeric sequence. The WT type strain Y-12630 was used for normalization. Twofold serial dilution of gDNA was prepared in TE buffer and boiled in 200 μl of denaturation buffer (0.4 M NaOH, 10 mM EDTA pH 8.2) for 10 min. After snap cooling on ice, DNA was vacuum-spotted using a Bio-Dot SF blotting system (BioRad) onto a Hybond-XL membrane pre-equilibrated with sterile H_2_O. Wells were washed with 0.4 M NaOH. The membrane was rinsed in 100 ml of 2x SSC buffer for 5 min, UV-crosslinked and hybridized with probes specific for telomeric sequences, and the TelKO element (20-mer TelKO-A: 5′- TGGGGTACGAGAAAATGTTG -3′). Hybridization was done at 45° ON and was followed by washing at 50° for 15 min, twice as described above. The signals were quantified using QuantityOne software (BioRad), normalized against λ DNA and then the value of WT DNA (WT DNA was set as 100%). Relative percentage values were plotted.

### DNA isolation and telomere-PCR

Cells were harvested at OD_600_ = 0.8, resuspended in 4 ml of SEB buffer (1 M sorbitol, 0.1 M EDTA and 14 mM β-mercaptoethanol) and incubated with 50 μL Zymolyase 20T at 37°, 60 rpm. The spheroplasts were harvested at 3 000 xg for 5 min and then incubated with 0.25 mg/ml proteinase K (Roche) in EDS buffer (50 mM EDTA, 0.2% SDS) at 37° for 1 h. Then 2 ml 3 M potassium acetate was added and incubated on ice for 1h. DNA was precipitated by adding an equal volume of isopropanol, rinsed in 2 ml 70% ethanol and resuspended in 0.5 ml TE buffer for ON. After RNase A incubation DNA was phenol-chloroform-extracted. DNA was precipitated by adding 1/10 volume of 5M NH_4_Ac and then 2.5 volume of 95% ethanol. Finally DNA was washed in 1 ml 70% ethanol and dissolved in 10 mM Tris-HCI, pH 8.0 at 4°.

To C-tail the genomic DNA (gDNA), 200 ng gDNA was denatured in 50 mM potassium acetate, 20 mM Tris acetate, 20 mM magnesium acetate, 0.25 mM cobalt chloride and 0.1 mM dCTP at 98° for 10 min, tailed with 0.2 U Terminal Transferase (NEB) at 37° for 30 min and heat-denatured at 70° for 10 min. Non-tailed DNA was prepared the same as tailing but terminal transferase was replaced with H_2_O. For the PCR reactions, 25 μl of 1 μM forward primer (Subtel-F3), 1 μM reverse primer (poly(G)_18_), 8 ng C-tailed or non-tailed gDNA, was mixed with 25 μl JumpStart REDTaq Ready Mix Reaction Mix (SigmaAldrich). PCR amplification was initiated at 95° for 2 min, then carried out using 45 cycles of 95° for 30 s, 64° for 30 s, 72° for 3 min, and final extension 72° for 5 min. Primers used in telomere PCR:

Subtel-F3, 5′-TGGTTGTAATATTGGAATTATTTTGTAATTCTAGTTCCCCGTTGGG-3′; poly(G)_18_, 5′- TGCTCCATACATTACTTAT(G)_18_ -3′. PCR products were separated on a 0.8% agarose gel, gel extracted and sequenced.

### Data and reagent availability

Strains are available upon request. The authors affirm that all data necessary for confirming the conclusions of this article are represented fully within the article and its tables and figures. Supplemental material available at FigShare: https://doi.org/10.25387/g3.9632294.

## Results

### Telomerase-negative cells proliferate without a growth crisis

Telomere elongation by telomerase is the most common way to maintain telomeres, in order to sustain genome integrity and thus cell proliferation. In *S. cerevisiae*, telomerase is expressed constitutively and deletion of any sub-component of telomerase leads to a gradual telomere shortening (Lundblad and Szostak 1989; Singer and Gottschling 1994; Lendvay *et al.* 1996). The telomere attrition leads to replicative senescence within 50-100 cell divisions, as observed by a severe decrease in cell proliferation and small colonies with rough edges. However, a low amount of cells may survive this crisis, and are termed “survivors” (Lundblad and Szostak 1989; Lundblad and Blackburn 1993; McEachern and Blackburn 1995). With the aim to investigate telomere length maintenance in the absence of telomerase in *N. castellii*, we disrupted the *TLC1* gene, encoding the telomerase RNA subunit that provides the template for telomerase elongation. We previously inserted the kanMX3 marker gene to disrupt the *TLC1* in the diploid *N. castellii* strain Y235 (described in (Astromskas and Cohn 2009)). Five heterozygous transformants (*TLC1/tlc1*::*kanMX3*) were sporulated and ten asci were microdissected into four haploid spores (tetrad) (Figure S1A). Replica-plating onto YPD-geneticin plates showed that each tetrad contained two wild-type spores (WT; *TLC1*) and two knockouts (KO; *tlc1*::*kanMX3*) (Figure S1A). The genotype of the knockout strains was verified by PCR amplicon size evaluation, using primers annealing up- and downstream of the *TLC1* gene, respectively, and the *tlc1*^-^ genotype was further confirmed by Southern blot hybridization (previously described in (Astromskas and Cohn 2009)).

To verify the loss of telomerase activity, we performed *in vitro* telomerase assays of extracts made from both WT and *tlc1*^-^ strains (Figure S1B). We tested telomerase activity of all four spores of two tetrads, using the primer extension assay with a 16 nt long telomeric DNA primer. The primer extension assay is highly sensitive and displays each nucleotide added onto the primer (Cohn and Blackburn 1995). Telomerase extracted from WT spores elongated the primer by multiple additions of the telomeric repeat, showing the characteristic 8 nt repeat pattern (Figure S1B, lanes 1 and 4). Since the catalytic activity of telomerase is dependent on the *TLC1* RNA subcomponent, disruption of the *TLC1* gene would eliminate the telomerase activity. As expected, extracts from the KO spores did not show any extension of the primer, thus verifying the knockout of telomerase activity (Figure S1B, lanes 2-3).

**Figure 1:**
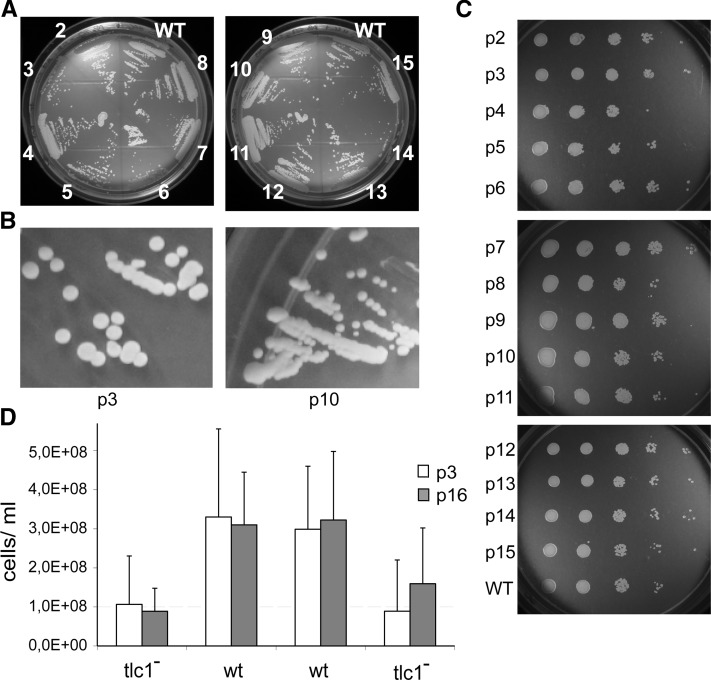
Telomerase-deficient *N. castellii* strains do not display any senescence phenotype. (A) Growth analysis of a diploid knockout strain (*tlc1*^-^/*tlc1*^-^) on YPD plates. The knockout strain and the isogenic wild-type (WT) strain from the same tetrad were continuously streaked onto YPD plates for ∼300 generations. The different passage numbers are indicated. The initially haploid spores switch into diploid cells already in p1. (B) Close-up of colonies from passages 3 *vs.* 10 (p3, p10). (C) Yeast spot test assay of passages 2-15 (p2-p15) and isogenic WT, 10-fold dilutions. (D) Growth assays in liquid YPD media. Colonies from p3 and p16 were inoculated into liquid YPD media at a final concentration of 5 × 10^5^ cells/ml and the cell density was counted after 24 hr. Each column represents the mean value of ten independent experiments. Bars denote standard deviation.

Immediately after germination of the spores, the *tlc1*^-^ KO colonies (correspond to p1) did not exhibit any difference in growth rate and colony morphology when compared to isogenic WT colonies (Figure S1A). Both WT and *tlc1*^-^ KO colonies formed bright and smooth colonies on the germination plates. In *S. cerevisiae*, telomerase-negative strains initially have WT-like colony shape and growth rate, but after continued propagation on plates they display senescence, typically at 80-100 generations (Lundblad and Szostak 1989; Lundblad and Blackburn 1993). To detect any senescence phenotype of our *N. castellii* telomerase-negative strains, we performed passages of the *tlc1*^-^ strains on YPD plates, re-streaking plates every two days. In total 16 *tlc1*^-^ strains were streaked for 8 passages (p8 >160 generations), and four of these strains were streaked for a total of 15 passages (p15 >300 generations). Unexpectedly, we did not observe any senescence, neither any severe growth defect, nor any major colony morphology changes (Figure 1A and B, Figure S1C). A spot test assay was performed on serial dilutions of cell cultures from passages 2 to 15 (p2-p15), which confirmed that all passages showed a very similar growth capacity (Figure 1C). However, in spite of a generally good growth ability, a slightly slower growth rate was observed during the passaging on plates, where *tlc1*^-^ strains required around half a day longer incubation time than WT cells to reach roughly the same colony size. To further investigate this, we performed a growth assay in liquid YPD media. Cultures of cells from passage 3 (p3) and 16 (p16) were inoculated to a concentration of 5 × 10^5^ cells/ml and the cell density was counted after 24 hr of incubation. The results showed that *tlc1*^-^ cells exhibit a slightly lower overall growth rate compared to isogenic WT cells (Figure 1D).

Even though we did not detect any widespread colony morphology defects, we noticed that the *tlc1*^-^ strains showed a population of small size colonies (0.5-1 mm) that differed noticeably from the WT colony size (1-3 mm). The colony size was not stable, since an ancestor with WT colony size generated both big and small colonies in next generations, and vice versa. Only rare colonies showed an uneven colony shape (“rough-edged”). Since the Y235 strain is able to switch mating type, the strains will eventually be able to mate and become diploid. Flow cytometry analysis confirmed that the majority of the cells in a culture became diploid already in p1, both KO and WT strains (data not shown). Since the mating occurs between isogenic cells, the strains become homozygous for the knockout allele (*tlc1^-^/tlc1*^-^). In conclusion, our results show that the proliferative capacity of diploid *N. castellii* strains is only mildly affected by the knockout of telomerase.

### Haploid N. castellii *tlc1*^-^ strains do not show any senescence phenotype

To investigate whether haploid telomerase-negative yeast strains would show the same survival capacity as the diploid, we used the same kanMX3 marker gene strategy to disrupt the *TLC1* gene in two stable haploid *N. castellii* strains (YMC48 and YMC63)(Karademir Andersson *et al.* 2016). Three knockout transformants were further analyzed, and the successful disruption was verified by PCR amplification using primers annealing 0.5-0.6 kb up- and downstream of the *TLC1* gene, respectively (Figure S2).

**Figure 2:**
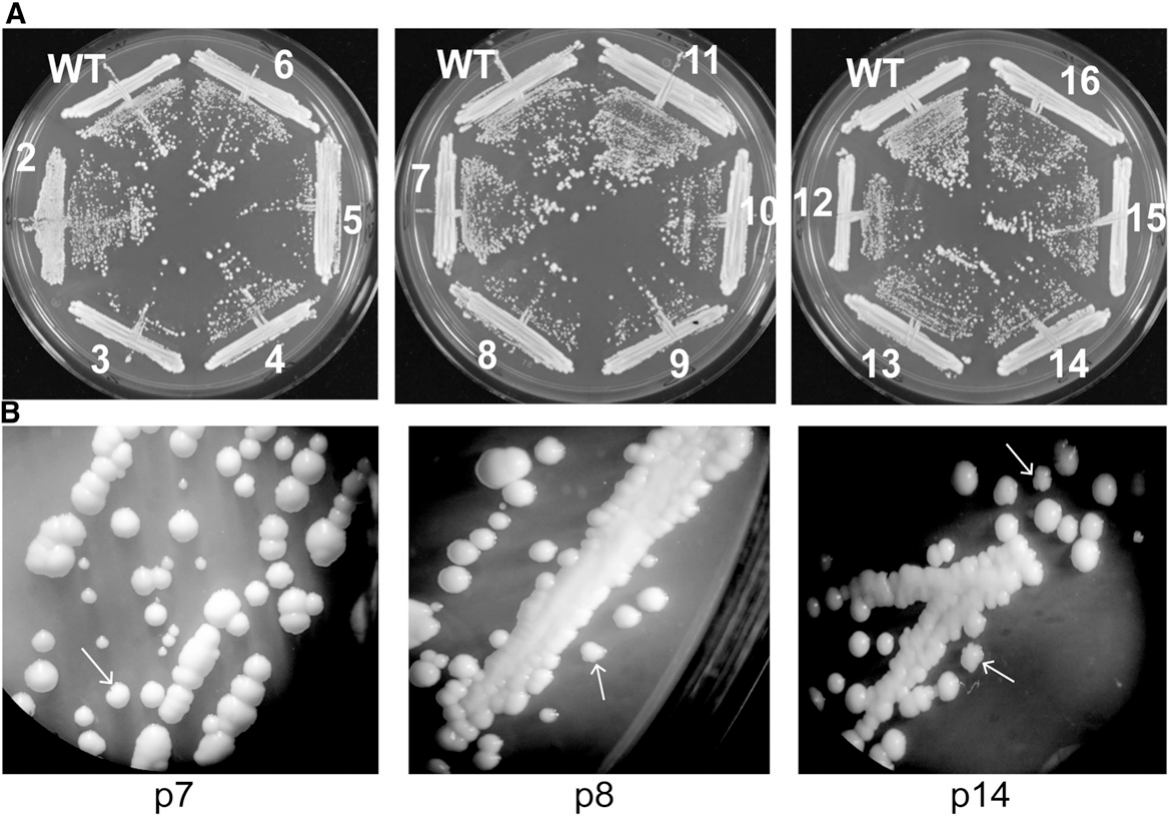
Haploid *tlc1^-^ N. castellii* strains do not display any senescence phenotype. (A) Growth analysis of the haploid knockout strain (*tlc1*^-^) on YPD plates. The knockout strain YMC481 and the wild-type (WT) parental strain (YMC48) were continuously streaked onto YPD plates. The different passage numbers are indicated. (B) Close-up of colonies from p7, p8 and p14. Arrows indicate colonies showing a minor rough-edge phenotype.

To analyze the growth ability, we passaged all three haploid *tlc1*^-^ strains on YPD plates for a total of 16 times, re-streaking every two days (Figure 2A). Similar to the diploid strain, we did not observe any senescence or severe abnormalities in colony morphology. However, haploid *tlc1*^-^ strains grew slightly slower than both their parental WT strains and the diploid *tlc1*^-^ strains, and required 1-2 days longer than the WT strain to form roughly the same colony size. Even though most colonies had WT colony morphology, we detected a small number of colonies with a slight uneven edge (rough-edged) (Figure 2B). The rough-edged colonies appeared mostly in passage 6-7 (p6-7) and subsequently decreased. Overall, colonies had WT morphology and only very few rough-edged colonies could be detected. Although small size colonies appeared, they also occurred in WT streaks, and the numbers did not significantly differ.

Together, our results show that neither diploid, nor haploid telomerase negative *N. castellii tlc1*^-^ strains exhibit any severe growth crisis when passaged for extensive numbers of generations. Furthermore, the lack of any marked senescence indicates a high frequency of telomerase-independent survivors.

### Telomerase-negative cells exhibit rapid telomere loss followed by dramatic incremental lengthening

**Figure 3:**
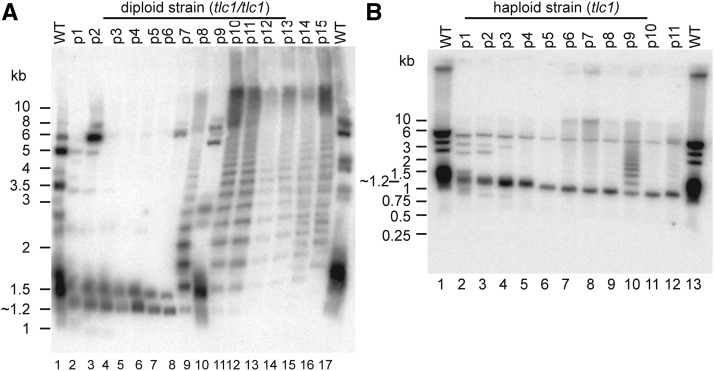
Telomere attrition is followed by generation of a novel type of ALT pattern. Terminal restriction fragment (TRF) assays of diploid (*tlc1^-^/tlc1*^-^) and haploid (*tlc1*^-^) survivors. Genomic DNA was digested with *Hind*III, separated on a 0.8% agarose gel and analyzed by Southern blot hybridization with a 16-mer telomeric sequence probe. (A) Diploid knockout survivors (*tlc1^-^/tlc1*^-^) from passage 1-15 (lanes 2-16) of YMC130, and WT DNA (lanes 1 and 17). In lane 10 (p9), a possible sample mix-up cannot be excluded. (B) Haploid knockout survivors (YMC481, *tlc1*^-^) from passage 1-11 (lanes 2-12), and WT DNA (YMC48) (lanes 1 and 13).

To study the telomere lengths in our telomerase-negative *N. castellii* strains, we performed Terminal Restriction Fragment (TRF) assays on colonies from each passage of the serial plate streaking procedure. Genomic DNA was extracted from each passage of five diploid homozygotes (*tlc1^-^/tlc1*^-^) and digested with *Hind*III enzyme. After electrophoresis and Southern blotting, telomeres were visualized by hybridization of the membrane with a 16-mer telomeric sequence probe (Figure 3A). *Hind*III-treatment of WT DNA produced several hybridizing fragments ranging from 1 kb to 6 kb, where some strongly hybridizing fragments indicate that several telomeres are collected together in the same size range (Figure 3A, lanes 1 and 17). The signal at ∼1.5 kb showed the smeariness that is characteristic of telomere fragments, which is due to the length variations between individual telomeres in the cell, as well as in a population of cells. In the *tlc1*^-^ strains, the same hybridizing bands were visible in the first few passages (p1-p3), but were observed to fade in signal with increasing number of passages. Moreover, the bands were gradually decreasing in size, hence indicating a gradual telomere shortening. Strikingly, most of the bands above 2 kb totally disappeared after p3 (corresponding to ∼75 generations) and the only remaining telomere signals in p4-p6 were found in two residual bands of approximately 1.2 and 1.5 kb length (Figure 3A, lanes 5-8). Interestingly, a dramatically changed banding pattern appeared in later passages (typically in p7-9), constituted by several bands of increasing size, like a ladder (Figure 3A, lane 9). The respective fragments of this ladder pattern showed an incremental size increase of approximately 300 bp, indicating that the ladder pattern may be formed by a repeated element. In combination with the appearance of the ladder pattern, the two residual 1.2/1.5 kb bands disappeared. It is interesting to note, that although the ladder pattern in Figure 3A changes somewhat in the relative strength of the bands, it is present in all of the following passages (except in p9) (Figure 3A, lanes 9-16). However, the ladder pattern occurred stochastically in the different strains (Figure S2C), and even varied in appearance between different preparations of the same strain.

The incremental ladder pattern exhibits an interesting profile. To investigate its characteristics, we hybridized the same membrane from above with a telomeric probe of the complementary strand, *i.e.*, the AC-rich strand (data not shown). This revealed the exact same TRF pattern as with the TG-rich telomeric probe. Since hybridization to both telomeric strands produced the same hybridizing fragments, we conclude that the *Hind*III-fragments of the ladder pattern contain double-stranded telomeric DNA.

Next, we wanted to investigate telomere attrition in the haploid *tlc1*^-^ strains. We made TRF assays of genomic DNA extracted from the three haploid *tlc1*^-^ strains using the same *Hind*III digestion (Figure 3B and S2D). Consistent with the diploid, the haploid WT DNA exhibits several strongly hybridizing fragments between 1-6 kb. In the first three passages a gradual telomere shortening is visible by the shortening of the fragments, and eventually the disappearance of most of them (Figure 3B, lanes 2-4). Notably, most of the remaining signal then appeared in a short defined fragment of ∼1.2 kb that remains through all the subsequent passages. Strikingly, a similar ladder pattern as previously seen in the diploid *tlc1*^-^ strains was observed in p9 (Figure 3B, lane 10). However, in a longer exposure, a faint ladder pattern can be visualized also in p6-p11, thus indicating a similar ladder pattern signal profile as in the diploid. In contrast to the diploid strain, however, the short fragment of ∼1.2 kb is still visible and has a strong hybridization signal through all passages in the haploid *tlc1*^-^ strain (see also Figure S2D).

In summary, both haploid and diploid *tlc1*^-^ knockout strains of *N. castellii* experience a rapid progressive shortening of telomeres. However, in later generation survivors, the TRF assay reveals a dramatic telomere phenomenon, where a ladder pattern is generated by band sizes increasing by ∼300 bp increments. The fact that the ladder pattern includes double-stranded telomeric sequences suggests that it is produced by an alternative telomere lengthening mechanism. Intriguingly, this is a novel type of ALT lengthening pattern, differing from the Type I and Type II patterns previously described in *S. cerevisiae*.

### EST1 knockout confirms that lack of a major growth crisis is a general response when telomerase is inactivated in N. castellii cells

The lack of a detectable major growth crisis in our *tlc1*^-^ strains of *N. castellii* is in great contrast to the phenotype previously found in *S. cerevisiae* (Lundblad and Szostak 1989; McEachern and Blackburn 1996). We wanted to investigate whether this high survival frequency is a general mechanism in response to a telomerase lengthening deficiency. Therefore, we aimed to delete the *EST1* gene, which is encoding a protein sub-component of the telomerase holoenzyme. Notably, the discovery of the characteristic delayed senescence phenotype of telomerase-deficient strains was made in *S. cerevisiae **est1*Δ mutants and was termed the *est* (ever shortening telomere) phenotype (Lundblad and Szostak 1989).

We constructed an *EST1* deletion cassette with 45 bp homologous sequences (Figure S3A). In order to facilitate the correct targeting and gene replacement, we used the “split-marker” approach, in which three-way recombination occurs to simultaneously insert and assemble the *K. lactis URA3* marker gene (Reid *et al.* 2002; Karademir Andersson *et al.* 2016). The two cassettes overlap with a 452 bp complementary sequence within the *klURA3* marker gene. The deletion construct was transformed into a diploid *ura3*^-^ strain (Y235) and six transformants were selected on drop-out plates lacking uracil. Deletion of the *EST1* gene was verified by PCR, using primers annealing 0.5 kb up- and downstream of the *EST1* gene, respectively. Subsequently, four correct transformants were sporulated and one ascus of each transformant was micro-dissected into tetrads (Figure S3B). The genotype of all germinated spores was verified by PCR amplification, where the replacement of the wild-type (WT) 3.2 kb band with a 2.5 kb knockout (KO) band showed that each tetrad contained two WT spores (*EST1*) and two KO spores (*est1*Δ::*URA3*) (Figure S3C). In order to further confirm the correct *EST1* deletion, we analyzed all four tetrads by Southern blot hybridization (Figure S3D). Hybridization with a *klURA3* probe, showed the expected 3 kb *BamH*I-generated band in the KO strains (*est1*Δ::*URA3*), which was lacking in the WT strains (*EST1*).

**Figure 4:**
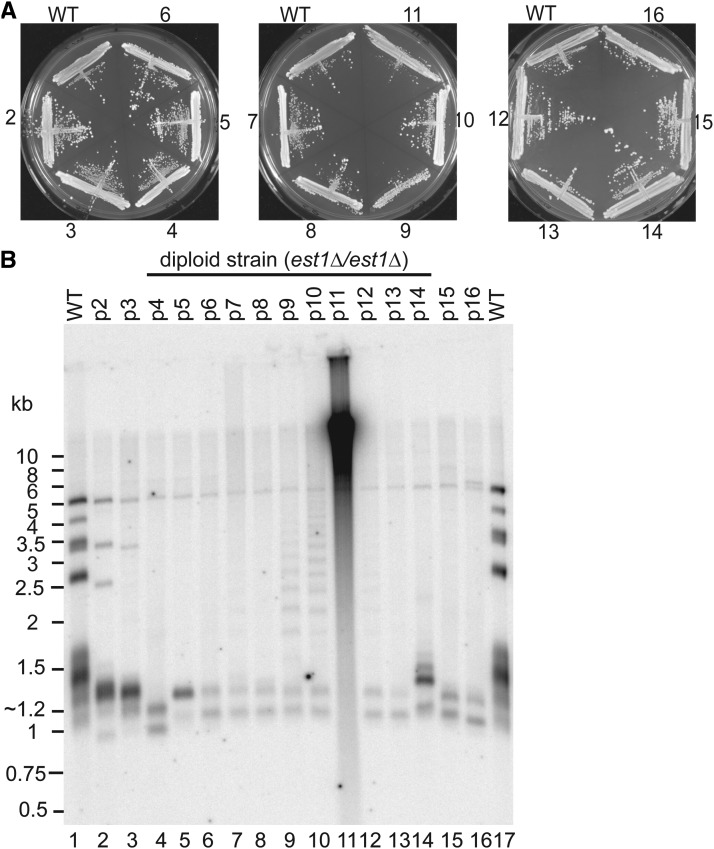
Deletion of *EST1* leads to the generation of the same type of ALT survivors as for the *tlc1*^-^ knockouts. (A) Growth analysis by continuous passaging of diploid (*est1*Δ/*est1*Δ) survivors and the wild-type (WT) strain onto YPD plates. Passage numbers are indicated. (B) TRF assay by *Hind*III digestion and Southern blot hybridization with a telomeric probe. Diploid (*est1*Δ/*est1*Δ) survivors from p2-p16 (lanes 2-16), and WT (lanes 1 and 17) YMC303 from tetrad 1 (Figure S3E).

The *est1*Δ spores were viable immediately after germination and they did not exhibit any major difference in growth rate and colony morphology when compared to isogenic WT spores (Figure S3B). Flow cytometry analysis showed that the majority of the cells in p1 cultures of both KO and WT strains are diploid (data not shown). We passaged all four tetrads 16 times on YPD plates every two days (Figure 4A). In accordance with the *tlc1*^-^ strains, we did not observe any senescence or any major abnormalities in colony morphology, and we could not detect any rough-edged colonies. Moreover, *est1*Δ knockouts had a similar growth rate on YPD plates and they showed a WT colony size after two days incubation. Taken together, these results show that bypassing the cellular senescence without a major growth crisis is a general phenotypic response in telomerase-negative *N. castellii* strains.

### The ladder pattern is a general phenomenon in N. castellii ALT cells

In order to determine whether telomerase-negative *N. castellii* cells use a common ALT pathway to maintain telomeres, we performed TRF assays of two of the diploid *est1*Δ strains derived from separate tetrads (Figure S3B, tetrad 1 and 2). DNA extracted from several passages was analyzed in the same way as described above for the *tlc1*^-^ strains (Figure 4B and Figure S3E). Intriguingly, we observed a similar rapid telomere attrition, which was then followed by generation of the ladder pattern phenomenon in later passages. As before, most of the original 1-6 kb telomeric bands disappeared after passage 4, leaving two very short bands at ∼1.2 kb and ∼1.4 kb that are kept throughout a majority of the passages (Figure 4B, lane 6). The ladder pattern appears strongly in p9-p10, but can be observed as faint signals also in p7-p8 (as well as p12 in Figure 4B and p11 in Figure S3E). Strikingly, in Figure 4B, p11 exhibits an extremely high signal, which is collected into a high molecular weight smear (Figure 4B, lane 11). Since some signal is also trapped in the well, it indicates the presence of extremely long DNA fragments that are not resolved in the gel. It is therefore tempting to speculate that the telomeres in the p11 cell population are super-elongated.

Taken together, the telomere analyses of *N. castellii* telomerase-negative cells reveal overall similar results for the *tlc1*^-^ and *est1*Δ strains. Both knockout mutants underwent a very rapid progressive telomere shortening, followed by telomere rearrangement and lengthening. Two different TRF assay patterns are generated, that show distinct and characteristic profiles. The pattern appearing in early generations constitutes mainly short hybridizing fragments of ∼1.2 kb and ∼1.4 kb. In contrast, in the later generations a striking ladder pattern emerges with incrementally longer fragments reaching sizes even beyond 10 kb. The ladder pattern appears transiently, and may stay on or disappear in the following passages, indicating stochastic ALT lengthening by specific repeated elements. In conclusion, a similar maintenance of telomeres was observed in both *tlc1*^-^ and *est1*Δ mutants, indicating a general ALT response in the absence of a functional telomerase enzyme. Moreover, the absence of a major growth crisis suggests that this ALT mechanism is quickly and easily activated in *N. castellii* telomerase-negative cells.

### Telomerase-negative cells maintain linear chromosomes

**Figure 5:**
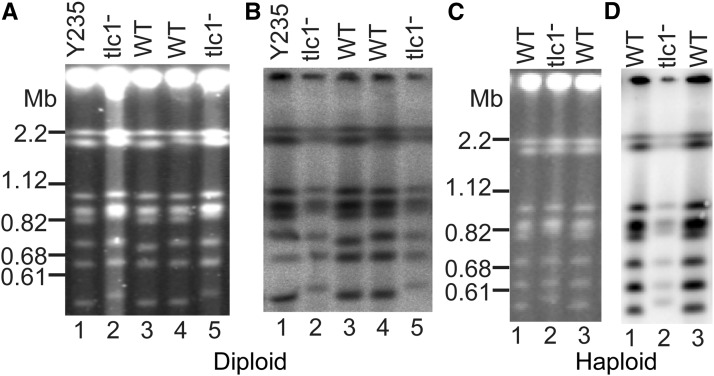
*N. castellii* ALT cells maintain linear chromosomes. Separation of whole chromosomes by pulsed field gel electrophoresis. (A, B) Diploid (*tlc1^-^/tlc1*^-^) strains. Two diploid knockouts (*tlc1^-^/tlc1*^-^) from p9 (lanes 2 and 5), and two isogenic wild-type (WT) strains obtained from the same tetrad (YMC121-124) (lanes 3-4). Y235 is the parental strain for the diploid KO (lane 1). (C, D) Haploid (*tlc1*^-^) strain YMC481 from p5 (lane 2), and its WT parental strain YMC48 (lanes 1 and 3). (A and C) Gels stained with EtBr. (B and D) Southern blot and hybridization of the membranes with a telomeric probe.

The fission yeast *Schizosaccharomyces pombe* has been shown to produce telomerase-negative survivors by using chromosome circularization (Nakamura *et al.* 1998). To investigate whether *N. castellii tlc1*^-^ cells still maintain linear chromosomes, we performed Pulsed Field Gel Electrophoresis (PFGE), as only linear chromosomes will enter the PFGE gel (Figure 5). To analyze clearly established survivors, we chose *tlc1*^-^ survivors from passage 9. As positive controls for linear chromosomes, we analyzed karyotypes of the parental strain and two isogenic WT strains obtained from the same tetrad, which all showed eight chromosomal bands in the PFGE (Figure 5A, lanes 1 and 3-4). As *N. castellii* was previously shown to have 10 chromosomes in the whole genome sequencing approach, probably some of the chromosomes are not resolving on the PFGE gel (Cliften *et al.* 2006; Gordon *et al.* 2011; Karademir Andersson and Cohn 2017). This notion is supported by the variation in signal intensity of the bands, suggesting that some bands include overlapping chromosomes. This is further supported by previous karyotyping experiments, where PFGE displayed eight or nine bands on the gel (Petersen *et al.* 1999). Notably, the chromosomes of the *tlc1*^-^ strains were resolved into the same eight bands as WT, thus showing that *tlc1*^-^ survivors maintain linear chromosomes (Figure 5A, lanes 2 and 5).

Next, we investigated the presence of telomeric DNA in survivor chromosomes, by performing a Southern blot of the PFGE gel and hybridization with the telomeric probe (Figure 5B). All chromosome bands of the WT strains strongly hybridized with the telomeric probe (Figure 5B, lanes 1 and 3-4). Similarly, all the bands of the knockouts (KO) exhibited hybridization signal, showing that all chromosomes indeed contain telomeric DNA. However, as expected, a lower signal intensity was obtained in the KO chromosomes, thus confirming the loss of telomere sequences.

PFGE analysis of haploid *tlc1*^-^ KO cells (passage 5) showed the same result as for the diploid cells (Figure 5C). The same eight chromosomal bands were visible both in the haploid parental WT strain and the KO strain. Similarly, a telomeric hybridization signal was observed for all bands, although the KO gives a lower signal intensity (Figure 5D). Notably, since the Ethidium Bromide stained PFGE gel showed a comparable signal intensity for the WT and KO strains, the lower hybridization signal indicates a quite high loss of telomeric DNA sequences in this KO strain. Interestingly, the smallest chromosome was observed to shift upwards in the KO strains. However, we were not able to correllate this phenomenon to the survivors, since we have observed variations in the migration of this chromosome.

In conclusion, our results show that telomerase-deficient *N. castellii* survivors are able to maintain linear chromosomes, and they do not exhibit any gross chromosomal changes. Notably, even though a loss of telomeric DNA is observed, all chromosome bands still show some remaining telomeric sequences, indicating that at least one of the telomeres of each chromosome contains some telomeric DNA.

### The ALT mechanism generates distinct repeats that are terminally located

**Figure 6:**
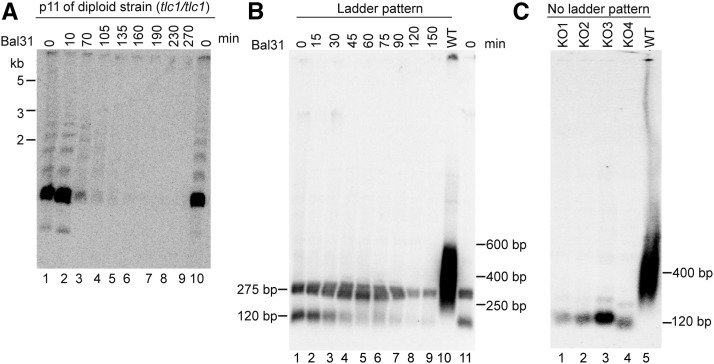
Bal31 assays show that fragments of the ladder pattern are terminally located. (A) Diploid (*tlc1^-^/tlc1*^-^) survivors from p11. DNA was cleaved with Bal31 for increasing periods of time and then treated as in the TRF assay, using *Hind*III. (B) Bal31 assay using *Dde*I of diploid (*tlc1^-^/tlc1*^-^) survivors from p11 having the ladder pattern (lanes 2-9). *Dde*I generates two bands of ∼275 bp and ∼120 bp, respectively (lanes 1 and 11; 0 min). WT DNA shows a ∼400 bp smear (lane 10). (C) TRF assay using *Dde*I of four independent colonies of diploid (*tlc1^-^/tlc1*^-^) survivors from p11, which do not generate the ladder pattern (lanes 1-4), and WT (lane 5). These survivors lack the ∼275 bp band previously observed in the ladder pattern survivors.

The ladder pattern that was observed in the late passages of all the strains, implies that long repeats of a specific length are added to the telomeres in the survivors. To address whether the DNA fragments in the ladder pattern are terminally located on the chromosomes, we performed assays with the exonuclease Bal31, which cleaves dsDNA from the terminal ends (Figure 6A). In WT control DNA, cleavage with Bal31 exonuclease leads to a decreasing size of telomeric fragments with increasing incubation time, thus indicating their terminal location (Fridholm *et al.* 2013). Here, gDNA from a diploid *tlc1*^-^ strain (p11) was incubated with Bal31 for increasing periods of time, followed by the TRF assay procedure, *i.e.*, digested with *Hind*III, blotting and hybridization with a telomeric probe. The telomeres of this strain showed a clear ladder pattern (Figure 6A, lanes 1 and 10). Intriguingly, these ladder pattern fragments were sensitive to Bal31 treatment, as shown by a progressive shortening in size and eventually disappearance (Figure 6A, lanes 2-9). Thus, we could conclude that the ladder pattern fragments are terminally positioned on the chromosomes, which indicates that they are products of the ALT lengthening mechanism.

To analyze the structural change in the telomeres in more detail, we wanted to use an enzyme that cleaves more closely to the terminus than *Hind*III, to release a shorter and potentially more homogeneous terminal fragment. *Dde*I was found to collect all of the WT telomeres into fragments centered around ∼400 bp (Figure 6B, lane 10). Due to the variation in telomere length, the Southern blot hybridization shows an expected smeary pattern, ranging between 250-600 bp. Thus, the *Dde*I target site is situated very close to the ∼250 bp telomeric sequences. Remarkably, the ALT strain that previously showed the *Hind*III ladder pattern generated a very different result using *Dde*I, with two distinct telomeric fragments appearing at ∼275 bp and ∼120 bp, respectively (Figure 6B, lanes 1 and 11). Thus, all the previously observed *Hind*III ladder pattern fragments are collected into two distinct bands by *Dde*I. This indicates that the ALT telomeres exhibit a total reorganization where the telomeric sequences are incorporated into longer repeated units.

To determine whether these two distinct *Dde*I-fragments originate from sequences located terminally on the chromosomes, we performed a Bal31 assay (Figure 6B). The ∼120 bp fragment was sensitive to Bal31 treatment, as it gradually decreased in signal and totally disappeared after two hours of incubation (Figure 6B, lanes 2-9). Hence, we conclude that the ∼120 bp *Dde*I-fragment is terminally located. On the other hand, the hybridizing fragment of ∼275 bp was more resistant to Bal31 digestion. Its signal was only slightly diminished after two hours, which indicates more internally located fragments or repeated elements that are present in long arrays (Figure 6B, lanes 2-9).

Since the total telomeric signal is collected into the two *Dde*I fragments, it suggested that at least one of them would also be part of the *Hind*III ladder pattern. In order to test this hypothesis, we wanted to analyze gDNA extracted from ALT cells that are not producing any *Hind*III ladder pattern. Indeed, the ladder pattern is observed to occur stochastically and appears at a low frequency, and even two colonies from the same streak may differ regarding the presence of the ladder pattern. The *Hind*III-digested gDNA of four independent colonies from the same plate streak of the diploid *tlc1*^-^ strain (p11) were analyzed by TRF assay and the absence of the ladder pattern was confirmed (data not shown). We then performed the TRF assay using *Dde*I on these particular gDNA. As previously, the telomeric fragments of the WT DNA were collected into a smeary region at ∼400 bp with the *Dde*I digestion (Figure 6C, lane 5). In contrast, the telomerase KO survivors generated a single prominent hybridizing fragment of ∼120 bp. Notably, the ∼275 bp band previously observed in the ladder pattern survivors was absent in these samples (Figure 6C, lanes 1-4). Therefore, we conclude that the ∼275 bp fragment is clearly coupled to the ladder pattern, and each distinct telomeric fragment in the ladder pattern should contain at least one copy of this particular element.

### Telomerase-negative cells maintain the telomere-adjacent subtelomeric region and short telomeric sequences at chromosome ends

**Figure 7:**
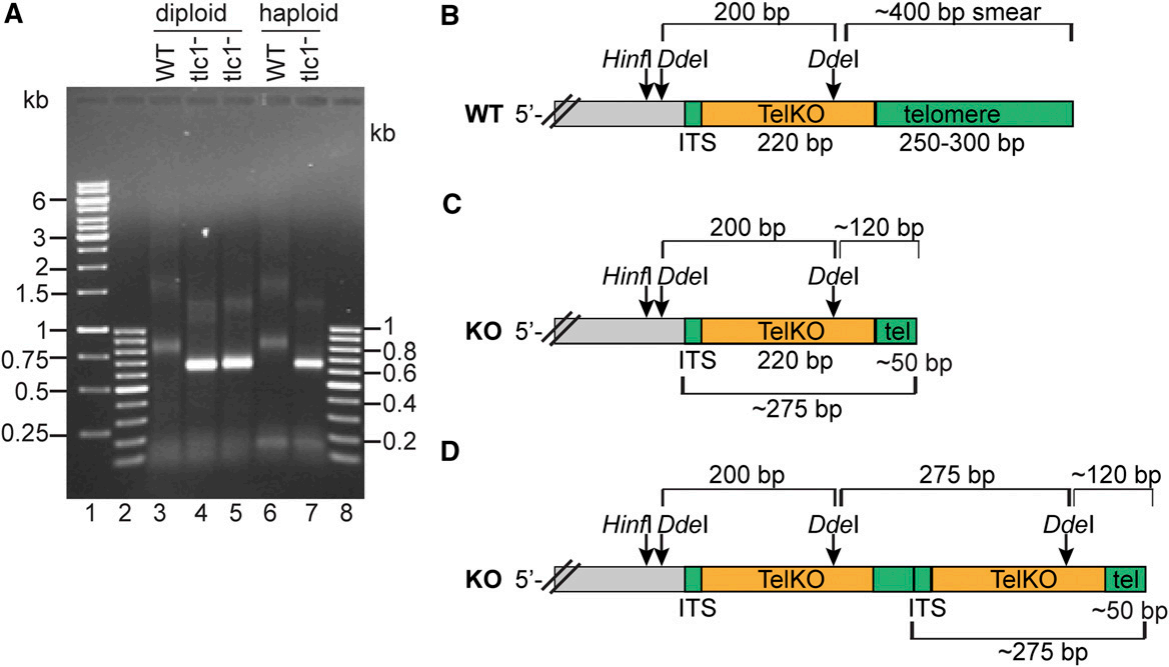
ALT cells maintain the same overall terminal DNA structure as WT cells, but have shorter telomeric DNA sequences. (A) Telomere-PCR analysis of C-tailed genomic DNA, WT diploid (lane 3), WT haploid (lane 6), diploid *vs.* haploid *tlc1*^-^ survivors (lanes 4-5 *vs.* 7). KO DNA both with (lane 4) and without (lane 5) a ladder pattern was analyzed. DNA size markers (lanes 1, 2, 8). The respective ∼850 bp *vs.* ∼650 bp bands of the WT and *tlc1*^-^ samples were gel-extracted and sequenced. (B and C) Schematic representation of the terminal telomere sequences in WT cells and *tlc1*^-^ ALT cells (KO). (D) Schematic showing the terminal structure of cells with ALT elongation (a ladder pattern strain). ITS, denotes the 9 bp interstitial telomeric sequences. TelKO, denotes the telomere-adjacent subtelomeric region flanked by ITS. The *Hinf*I and *Dde*I recognition sites are indicated, together with the fragment sizes that *Dde*I generates.

Next, we wanted to reveal what DNA sequence is present at the terminus of the ALT telomeres. To that end, we performed two separate approaches. First, a PCR based technique, the so-called telomere-PCR method (Forstemann *et al.* 2000; Wahlin *et al.* 2003; Wang *et al.* 2014), and second a direct telomere cloning procedure. In telomere-PCR, the ends of genomic DNA was C-tailed using terminal transferase and dCTP. PCR amplification was performed using a forward primer targeting a previously identified subtelomeric region and a reverse poly(G)_18_ primer annealing to the C-tail. On an agarose gel, PCR amplification of both haploid and diploid WT strains yielded a broad band of ∼850 bp (Figure 7A, lanes 3 and 6). In addition, a weak and smeared band of ∼1.6 kb could be observed. An additional weak ∼150 bp band was determined as a background since it also appeared in the non-tailed control gDNA (data not shown).

Telomere-PCR of the *tlc1*^-^ strains similarly produced two bands, but with a smaller size, ∼650 bp and ∼1.4 kb (Figure 7A, lanes 4, 5 and 7). Notably, the ∼650 bp band appears quite distinct in both the KO strain containing, and the one lacking a ladder pattern (Figure 7A, lanes 4 and 5, respectively). It is worth noting that the size difference of the most prominent bands in the WT and KO strains is 200 bp, which would correspond to the shortening of the telomere sequences. To analyze whether these products contain terminal sequences of the chromosome ends, we gel-extracted the ∼850 bp and ∼650 bp bands from WT and KO strains, respectively.

Sequencing by using various primers targeting the subtelomeric region revealed that both WT and *tlc1*^-^ KO strains contain the same 220 bp subtelomeric region, which we termed the TelKO element (Figure 7B, Figure S4A). In the WT telomeres, the TelKO element is residing just interior of the terminal telomeric DNA sequences. Thus, we have here determined the TelKO region to be the most telomere-proximal subtelomeric sequence. Notably, it contains a *Dde*I recognition site, which is the site that releases the ∼400 bp telomere smear (Figure 6B). Moreover, the TelKO element is flanked by a short 9 bp interstitial telomeric sequence (ITS), defining a boundary to the continued subtelomeric sequences. Strikingly, also in the ALT strains, the TelKO element is located just internally of a telomeric sequence that is terminally located. However, the stretch of telomere sequence is shorter, around 50 bp (Figure 7C, Figure S4A).

The telomere-PCR of the survivors was performed on gDNA extracted from p11, and thus represent clearly established ALT strains. Since we could identify the C-tail in our sequencing results, we could determine that the telomere sequence stretch of ∼50 bp was indeed terminally located. Even though the exact number of basepairs in the telomere sequence differed in the separate sequencing samples, the TelKO element was invariably located interior of it (Figure 7C). We also obtained the same sequence organization from other primer combinations in the telomere-PCR, thus confirming our results (data not shown). Therefore, we conclude that the ALT cells indeed contain a short telomeric stretch (∼50 bp) at the very end of the chromosome, and that the telomere-adjacent region (TelKO element) is also maintained.

Second, we performed a direct cloning of telomeres. Since we concluded that the *Dde*I fragments reside very close to the terminus, we set out to clone telomeres by constructing a sub-genomic library of *Dde*I-fragments. DNA extracted from survivors was digested with *Dde*I, separated on an agarose gel and the fragments with appropriate sizes were gel-extracted and cloned. *E. coli* clones were screened by colony hybridization with a telomeric probe and positive clones were analyzed further by plasmid purification and slot blot hybridization, resulting in three clones with a strong signal. Sequencing showed that the clones contained short stretches of telomeric sequences of different lengths (27 bp, 55 bp and 56 bp) (Figure S4B and S4C). Moreover, they all contained a part of the TelKO element (64-65 bp, termed TelKO-A). Two of the clones additionally shared another part of the TelKO element (TelKO-B), flanking the other side of the telomeric sequence. The organization of these cloned *Dde*I fragments, with telomeric sequences flanked by upstream and downstream parts of the TelKO element, indicate that they emanated from internal positions in the ALT-extended telomeres.

In summary, the two isolation approaches together revealed important features of the survivor telomeres. First, the knockouts still maintain telomeric sequences at the extreme termini, although drastically shortened. Second, the presence of ∼50 bp internally positioned telomeric sequences, which are flanked by the most terminal subtelomeric element (TelKO), indicates that telomeres are lengthened by repeated elements containing both telomeric sequences and the telomere-adjacent TelKO element. The assembly of the sequence information allowed us to construct a schematic model for the elongated array structure of the ALT telomeres (Figure 7D). The situation where two consecutive TelKO elements are flanking a ∼50 bp telomeric sequence creates two *Dde*I fragments containing telomeric sequences; the ∼275 bp internal fragment and the ∼120 bp terminal fragment (Figure 7D). These fragments correspond to the previously observed *Dde*I bands in the TRF assay, which therefore confirms the structure (Figure 6B). Significantly, this data suggests that the ladder pattern observed in the TRF assay is generated by the addition of ∼275 bp elements onto the ends of ALT chromosomes.

### ALT telomeres are extended with a specific ∼275 bp element containing both subtelomeric and telomeric DNA

**Figure 8:**
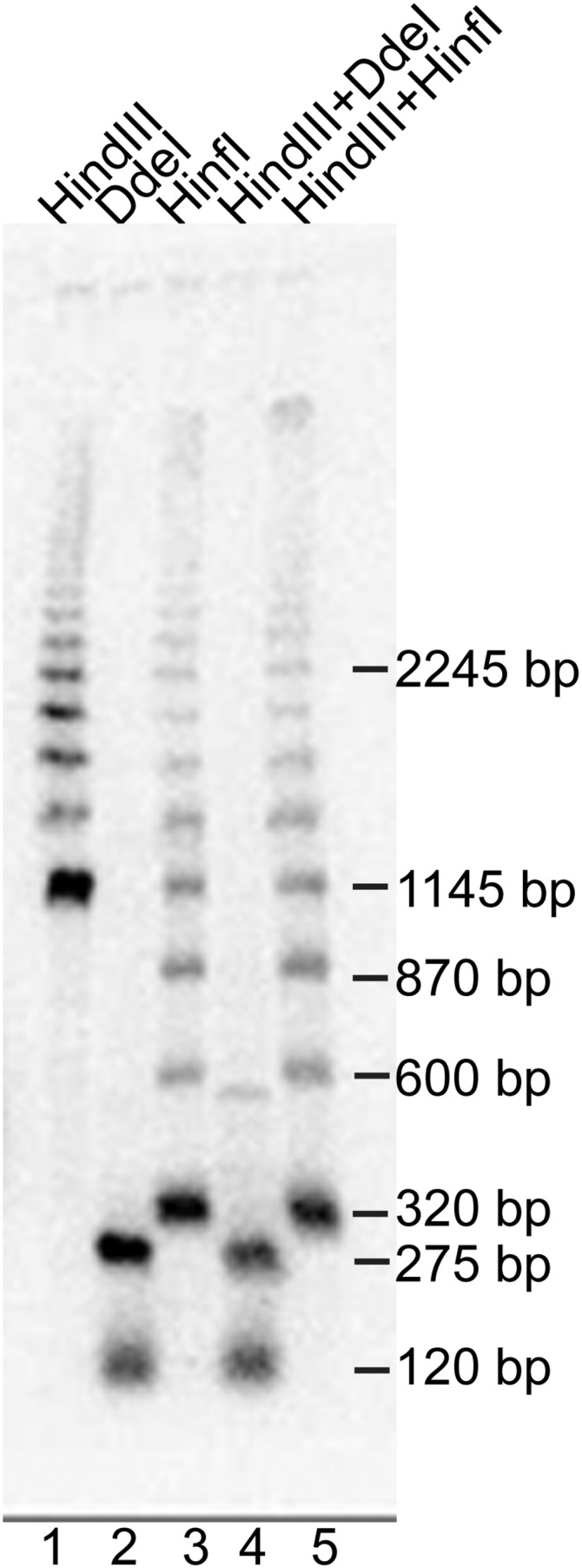
TRF assay using *Hinf*I and *Dde*I confirms that ALT cell telomeres are elongated by ∼275 bp TelKO elements. An ALT strain exhibiting the *Hind*III ladder pattern was digested with the *Hinf*I and *Dde*I enzymes in single *vs.* double digestions, as indicated. Note that *Hinf*I cuts more terminally than *Hind*III, thus shifting down the ladder pattern. The sizes of some bands in the *Hinf*I ladder are indicated, as well as the specific ∼275 bp *Dde*I band that is only observed in ALT strains having the ladder pattern, *i.e.*, that are elongated by TelKO elements.

The sequence analyses of the cloned telomere fragments suggested that the *N. castellii* ALT chromosomes are extended with ∼275 bp repeats containing the subtelomeric TelKO element and telomeric sequences. To further confirm the proposed structure and organization of the ALT telomeres, we made a TRF assay using various restriction enzymes (Figure 8). We selected an ALT strain exhibiting the characteristic ladder pattern when digested with *Hind*III, where the size of the bands is increasing in ∼275 bp increments (Figure 8, lane 1). The *Hinf*I site is located more terminally than the *Hind*III site, and is therefore expected to release shorter terminal fragments. As expected, *Hinf*I digestion is shifting down the ladder pattern into smaller fragment, although maintaining the same incremental steps, hence confirming that the repeats are terminally located (Figure 8, lane 3). In stark contrast, *Dde*I digestion of the ALT strain collects the telomeric signal into the two distinct bands of ∼275 bp and ∼120 bp, as shown previously (Figure 8, lane 2). Double digestions, further confirm this structure, since the more terminally located *Dde*I and *Hinf*I sites overrule the more internally located *Hind*III site (Figure 8, lanes 4-5).

To further confirm the subtelomeric structure, we rehybridized the membrane with a probe annealing to the TelKO element (Figure S5). As expected, the TelKO element hybridized to the ∼275 bp *Dde*I fragment of the ALT strain. Taken together, the specific band patterns obtained with various restriction enzymes in the TRF assay confirm the structure obtained from the telomere-PCR sequence data. Please see the Supplementary Figure S5 for the full description of the confirmation.

The addition of the long arrays of repeated ∼275 bp elements appears rather dramatic in the TRF assay, but may in theory be derived from a few lengthening events. To gain information regarding the extent of the elongation events, we quantified the amount of TelKO elements in the genome by slot blot hybridization. Serially diluted genomic DNA, both from ALT cells with a ladder pattern profile and without ladder profile was put on the membrane, together with the WT *N. castellii* Y-12630 (type strain) and hybridization controls (Figure S6). First, we performed hybridization of the membrane with the telomere probe, which showed that the amount of telomeric sequence is markedly decreased in the ALT cells compared to the WT. Moreover, a slight difference between the two types of ALT cells was observed (Figure S6). As in the TRF analysis, the ALT cells without a ladder profile showed a lower signal than the ladder profile cells, indicating that a slightly lower amount of telomere sequence is present in those cells that are not being elongated with the ∼275 bp element.

Next, we hybridized the membrane with a probe directed toward the TelKO region. Compared to WT, the TelKO hybridization signal was almost twofold higher in the ALT cells having the ladder pattern (Figure S6). In contrast, ALT cells without ladder profile showed a slightly lower content of the TelKO element compared to WT. Hence, although TelKO elements are added onto the ALT cell telomeres, the overall increase of elements is rather modest, thus indicating a limited elongation activity.

Taken together, our results show that late passages of *N. castellii* ALT strains contain ∼275 bp repeated elements including both telomeric sequences and the internal subtelomeric region just adjacent to it, the TelKO element (Figure 7D). The TelKO element is a part of the WT telomere structure, but is increasing in numbers in the ALT strains due to addition of several consecutive repeats. When several repeats are added to a single telomere, forming an array of repeats, this is visualized as a ladder pattern in the TRF assay. We suggest that the elongation event may be accomplished by the shortened telomere invading the subtelomeric region of another chromosome, to use it as a template to copy toward the end. The interstitial telomeric sequence (ITS) that is flanking the TelKO element would be able to act as a starting point for this event. In fact, the sequences from the cloned internal telomeric segments end in exactly the same permutation as the ITS, thus implying the alignment of the shortened telomere with the ITS in the initiation of the elongation process.

## Discussion

Genome stability in eukaryotes relies on the structural and the functional integrity of telomeres. The most widely adopted way to maintain telomeres is by utilizing the enzyme telomerase. However certain eukaryotic organisms can restore telomere length and thus chromosomal linearity in the absence of a functional telomerase (Lundblad and Blackburn 1993; McEachern and Blackburn 1996; Kass-Eisler and Greider 2000; Maringele and Lydall 2004; Jain *et al.* 2010; Xu and McEachern 2012). When telomerase fails to maintain telomeres, alternative mechanisms may be activated, so-called Alternative Lengthening of Telomeres (ALT). ALT pathways are well characterized in certain yeast species, including *S. cerevisiae*, *K*, *lactis*, *and S. pombe* (Lundblad and Blackburn 1993; McEachern and Blackburn 1996; Nakamura *et al.* 1998; Kass-Eisler and Greider 2000; Natarajan and McEachern 2002; Maringele and Lydall 2004; Jain *et al.* 2010; Xu and McEachern 2012). These studies demonstrate the presence of various ALT pathways that are activated in a context-dependent manner.

In this study, we investigated telomerase-independent telomere maintenance in the budding yeast *N. castellii*. Intriguingly, we found a novel ALT mechanism that provides a very efficient backup mechanism for telomere maintenance when telomerase is inactivated. In contrast to *S. cerevisiae* and *K. lactis*, telomerase-negative *N. castellii* cells do not display any major growth crisis and they maintain WT colony morphology (Lundblad and Szostak 1989; McEachern and Blackburn 1996). This undetectable growth crisis is a general response to loss of telomerase activity, as it occurs in both *tlc1*^-^ strains and *est1*Δ strains. Moreover, it is not dependent on the cell type, since both diploid and haploid *tlc1*^-^ strains proliferate over 300 generations without displaying any growth senescence. Thus, *N. castellii* tolerates loss of telomerase surprisingly well, and conveniently avoids cellular replicative senescence. Notably, lack of a prominent senescence has also been observed in *Candida albicans* telomerase KO mutants (Steinberg-Neifach and Lue 2006).

*N. castellii* ALT cells experience a rapid progressive telomere shortening and exhibit a reorganization of the telomeric sequences (Figure 3). Interestingly, in later generations they use a unique way to counteract telomere attrition. This novel ALT pathway elongates telomeres by addition of ∼275 bp repeated DNA elements that contain both subtelomeric DNA and telomeric sequences, termed TelKO elements. The elongation is observed as a striking ladder pattern in the TRF assay, showing the incremental lengthening by uniform TelKO repeats. It is noteworthy that these particular features differ from the Type I and Type II ALT mechanisms that were previously reported in *S. cerevisiae*. However, some conceptual similarity to the Type I amplification of subtelomeric Y’ elements can be noted.

Although the repeat addition generates a spectacular ladder pattern with a relatively high signal in the TRF assay, the quantification does not show any drastic increase in the amount of TelKO elements in the KO genome. This may indicate that the number of TelKO repeats added to each telomere is low, or alternatively, only a few telomeres may be elongated at each specific time point. Potentially, the TelKO arrays may be present only in a subset of the cells in a population. We further hypothesize that the array may be rather dynamic, with alternating lengthening and shortening of the repeat arrays.

The repeated TelKO elements contain the most telomere-proximal subtelomeric region, as well as a ∼50 bp stretch of telomeric sequence that are internally located in the repeat array. A homology search of the TelKO element in the *N. castellii* genome database did not generate any match. However, even though the whole *N. castellii* genome is sequenced, it lacks telomeric and terminal subtelomeric sequences (Cliften *et al.* 2006; Gordon *et al.* 2011). This further supports that the location of the TelKO elements is restricted to the distal subtelomeres. In accordance with previously proposed models for homology-directed ALT maintenance, the addition of TelKO elements may form via inter-telomeric replication where a single-stranded 3′ overhang of an eroded telomere would invade the duplex DNA of another telomere to use it as a copy template (Pickett and Reddel 2015). Intriguingly, we found evidence that the TelKO element sequences start at a 9 bp region of interstitial telomeric sequences (ITS) present within the subtelomere. This feature implies that the ITS is the target site for the invasion of a shortened telomere and hence functions as the specific initation site for the copying. We therefore propose that this specific subtelomere structure forms the fundament for the rescue of eroded telomeres, by forming the template for the elongation of the shortened telomeres with repeated TelKO elements.

On the other hand, we cannot exclude the possibility that the TelKO elements may be added by the “roll and spread mechanism”, which was demonstrated in telomerase-negative *K. lactis* strains (Natarajan and McEachern 2002; McEachern and Haber 2006; Xu and McEachern 2012). The repeated element would in such a case be templated by a telomeric circle (t-circle) that is generated by excision of a t-loop. In the *N. castellii* survivors, such a t-circle would however need to contain also the subtelomeric region. *K. lactis* telomerase KO cells transformed with DNA circles containing both telomeric repeats and non-telomeric sequences are indeed able to add alternating regions to telomeres. Interestingly, such alternating terminal structures are also present in *S. cerevisiae* Type I survivors, where subtelomeric Y’ elements are insterspersed with telomeric sequences. It is tempting to speculate about the possibility that different types of lengthening mechanisms may actually take place, which could be depending on the actual structure being present in individual telomeres at specific time points. It is interesting to note that the later passages show a maintained short telomere structure. Hypothetically, this could suggest an efficient copying of telomeric sequences from one short end onto another short end. Possibly, the short uniform telomeric repeats of *N. castellii*, in contrast to the irregular telomeric sequences in *S. cerevisiae*, could be allowing for this process. Moreover, the short length of the ITS may imply that the initial annealing of homologous sequences are rare events, while subsequent array extensions could be promoted by using the full TelKO element for annealing. Thus, the intitial process may be replaced by other subsequent mechanisms at individual telomeres where a TelKO element has been added.

Despite the telomeric erosion, telomerase-negative *N. castellii* cells maintain their linear chromosomes and lack abnormally sized chromosomes. Interestingly, our data show that they maintain a WT structural organization at the very ends of the chromosomes and a short stretch of telomeric sequences at the terminus. The short stretch of terminal telomeric sequence might avoid extensive chromosomal rearrangement through its association with telomere-specific proteins. We previously showed that *tlc1*^-^ KO cells do indeed have short 3′ overhangs that would provide binding sites for the Cdc13 protein (Rhodin Edsö *et al.* 2008; Fridholm *et al.* 2013). The Rap1 protein binds every 16 nt of duplex DNA and thus the 50 bp telomeric sequence of the mutant telomeres would be bound by 2-3 Rap1 molecules (Wahlin and Cohn 2002). Therefore, the telomere binding proteins may shield mutant telomeres from end to end fusions and thus inhibit massive chromosomal rearrangements.

In conclusion, our results show that *N. castellii* bypasses cellular senescence very proficiently and effectively after telomerase is disabled. The telomerase-negative cells proliferate without neither growth crisis nor severe growth defects. We propose that the specific subtelomere structure present in some telomeres forms the fundament for the rescue of eroded telomeres, by forming the copy template for the addition of repeated elements containing both telomere sequences and the adjacent subtelomere region onto shortened telomeres. This uniquely efficient ALT mechanism is a general response to telomerase dysfunction and is activated in both diploid and haploid cells. In essence, it provides the ability to maintain linear chromosomes and WT structural organization at the terminal ends of chromosomes. ALT cells preserve short telomeric tracts at their chromosomal ends, which might provide the necessary minimal protection and thus help to overcome the replicative limitations. As this ALT pathway displays novel characteristics, it will be highly interesting to further elucidate the underlying molecular mechanisms.
